# NEO212 induces mitochondrial apoptosis and impairs autophagy flux in ovarian cancer

**DOI:** 10.1186/s13046-019-1249-1

**Published:** 2019-06-07

**Authors:** Xingguo Song, Lisheng Liu, Minghui Chang, Xinran Geng, Xingwu Wang, Weijun Wang, Thomas C. Chen, Li Xie, Xianrang Song

**Affiliations:** 1 Shandong Provincial Key Laboratory of Radiation Oncology, Shandong Cancer Hospital affiliated to Shandong University, Shandong Academy of Medical Sciences, 440 Ji-Yan Road, Jinan, 250117 Shandong Province People’s Republic of China; 2 Key Laboratory of Animal Resistance Research, College of Life Science, Shandong Normal University, 88 East Wenhua Road, Jinan, Shandong People’s Republic of China; 3 School of Medicine and Life Sciences, University of Jinan, Shandong Academy of Medical Sciences, Jinan, Shandong People’s Republic of China; 4 Department of Clinical Laboratory, Shandong Cancer Hospital affiliated to Shandong University, Shandong Academy of Medical Sciences, 440 Ji-Yan Road, Jinan, 250117 Shandong Province People’s Republic of China; 5 Maternity & Child Care Center of Dezhou, Dongdizhong Street 835#, Decheng District, Dezhou, Shandong People’s Republic of China; 6 Departments of Neurological Surgery, and Pathology, University of Southern California, Los Angeles, California USA

**Keywords:** NEO212, Mitochondrial apoptosis, Autophagic flux, TFEB, Ovarian cancer

## Abstract

**Background:**

Temozolomide-perillyl alcohol conjugate (NEO212), a novel temozolomide (TMZ) analog, was previously reported to exert its anti-cancer effect in non-small cell lung cancer (NSCLC), and human nasopharyngeal carcinoma (NPC), etc.. In the current study, we intend to illuminate the potential anticancer property and the underly mechanisms of NEO212 in ovarian cancer cells.

**Methods:**

The cytotoxicity of NEO212 was detected by MTT, colony formation analysis and xenograft model. The proteins involved in cell proliferation, DNA damage, autophagy and lysosomal function were detected by western blots; mitochondria, lysosome and autophagosome were visualized by TEM and/or immunofluorescence; Apoptosis, cell cycle analysis and mitochondrial transmembrane potential were detected by flow cytometry. TFEB translocation was detected by immunofluorescence and western blot.

**Results:**

NEO212 has the potential anticancer property in ovarian cancer cells, as evidence from cell proliferation inhibition, G_2_/M arrest, DNA damage, xenograft, mitochondrial dysfunction and apoptosis. Importantly, we observed that although it induced significant accumulation of autophagosomes, NEO212 quenched GFP-LC3 degradation, down-regulated a series of lysosome related gene expression and blocked the autophagic flux, which significantly facilitated it induced apoptosis and was largely because it inhibited the nuclear translocation of transcription factor EB (EB).

**Conclusions:**

NEO212 inhibited TFEB translocation, and impaired the lysosomal function, implying NEO212 might avoid from autophagy mediated chemo-resistance, thus proposing NEO212 as a potential therapeutic candidate for ovarian cancer.

**Electronic supplementary material:**

The online version of this article (10.1186/s13046-019-1249-1) contains supplementary material, which is available to authorized users.

## Background

Ovarian cancer is the most lethal gynecological malignancy [1], which seriously threatens the health of women. Current treatment for ovarian cancer includes a combination of surgery and chemotherapy [2]. However, many patients carry poor prognosis even after long-term systemic therapy with cytotoxic agents [3]. Ever since the platinum-based chemotherapy was applied to clinic, the survival rate of women with ovarian cancer has had no obvious improvement for nearly 40 years, thus, there is a need to develop more effective agents to improve this situation.

Temozolomide-perillyl alcohol conjugate (NEO212), a novel temozolomide (TMZ) analog, is developed based on the conjugation of TMZ, a clinically applied alkylating agent, and perillyl alcohol (POH), a naturally occurring monoterpene that has been used orally for the treatment of a variety of cancers [4]. This novel compound has recently attracted continuous attention since accumulating data demonstrate that it exhibits stronger anti-cancer potency than either of its individual components (TMZ and POH) in several types of malignancy such as glioma [5], triple-negative breast cancer (TNBC) [6], non-small cell lung cancer (NSCLC) [7, 8] and human nasopharyngeal carcinoma (NPC) [9]. In this paper, we intend to illuminate the potential anticancer property of NEO212 in ovarian cancer cells.

Mitochondria as crucial organelles regulate cellular energy generation, calcium and redox homeostasis and apoptosis. Given that, targeting mitochondria in cancer cells is considered as an attractive therapeutic strategy [10]. To perform the cellular functions effectively, mitochondria continuously change their structure and morphology through protein machineries controlling fission and fusion process [11]. Fission is needed to create new mitochondria, but it can also facilitate apoptosis during high levels of cellular stress [12] such as chemical reagent treatment. This occurs almost simultaneously with two steps of apoptosis that involve mitochondria: translocation from the cytosol to mitochondria of the pro-apoptotic Bcl-2 family member BAX and Cytochrome C release [13]. Our previous studies have demonstrated that NEO212 was capable of inducing ROS accumulation, mitochondrial membrane potential collapse and mitochondrial apoptosis [7, 9], indicating mitochondria might be one of main targets of NEO212.

Damaged mitochondria can trigger apoptosis, it prevents cellular harm and is crucial for cell survival. An increasing number of researches shows that autophagy can give cancer cells resistant ability to apoptosis, whereas inhibition of autophagy causes apoptotic cell death [14] [15]. Understanding the crosstalk between apoptosis and autophagy in cancer cells is vital to find out new strategies for cancer therapy and improve the therapeutic efficiency. Hence, the ability of NEO212 to affect apoptosis and autophagic activity in ovarian cancer cells should be revealed. Previous studies have established the association between chemotherapy with TMZ and autophagy [16]. Interestingly, when autophagy was prevented at an early stage by 3-methyladenine (3MA), the antitumor effect of TMZ was suppressed, whereas bafilomycin A1 (Baf.A1) that prevents autophagy at a late stage by inhibiting lysosome acidification and its fusion with autophagosome, sensitized tumor cells to TMZ by inducing apoptosis, indicating the chemotherapy efficacy of TMZ depends on it induced autophagic flux status.

Terminally damaged mitochondria are sequestered in a double membrane vesicle (autophagosome), and then fuses with a lysosome, causing its contents to be degraded. The function of autophagosome and lysosome can be regulated by some transcriptional factors, such as transcription factor EB (TFEB). TFEB regulates lysosomal biogenesis by stimulating the expression of proteins involved in all steps of lysosome [17], is activated by an increased need for lysosomal activity or when lysosomal function is impaired [18]. Interestingly, TFEB is also involved in mitochondrial dysfunction and has been reported to regulate mitochondrial biogenesis via inducing the expression of major related master regulator genes [19], indicating that mitochondria and lysosomes share strong functional links that could play a fundamental role in both normal physiology and pathology [20].

In the current study, we aimed to reveal the potential anticancer property of NEO212 in ovarian cancer and the underlying mechanisms. We found that NEO212 exerted an antitumor effect in ovarian cancer, as evidence from cell proliferation inhibition, G_2_/M arrest, DNA damage, xenograft, mitochondrial fission and apoptosis. Importantly, we observed that NEO212 blocked autophagy flux although the number of autophagosomes was increased, which significantly facilitated it induced apoptosis and was largely because NEO212 inhibited the nuclear translocation of transcription factor EB (EB), and impaired the lysosomal function, thus proposing NEO212 as a potential therapeutic candidate for ovarian cancer.

## Methods and materials

### Cell lines and chemicals

Human ovarian cancer-derived cell lines SK-O-V3 and OVCAR-3 were purchased from American Type Culture Collection (ATCC, Manassas, VA, USA); A2780 were purchased from KeyGEN BioTECH (Jiangsu, China). A2780 and OVCAR-3 cells were grown in Dulbecco’s modified Eagle’s medium (DMEM, Thermofisher, Carlsbad, CA, USA), SK-O-V3 cells were grown in McCoy’s 5A (modified) Medium (Thermofisher), supplemented with 10% fetal bovine serum (Thermofisher) and antibiotics (penicillin/streptomycin, 100 U/ml, Beyotime, Beijing, China) at 37 °C in 5% CO_2_.

NEO212 and Perillyl alcohol (POH) were provided by Neonc Technologies, Inc. (Los Angeles, USA) and diluted with DMSO to make stock solutions of 100 mM. Temozolomide (TMZ), 3-methyladenine (3MA), Baflomycin A1 (Baf.A1), Earle’s Balanced Salt Solution (EBSS) (Sigma-Aldrich, Shanghai, China) were dissolved in DMSO or deionized water dependently; In all cases of cell treatment, the final DMSO concentration in the culture medium never exceeded 0.5%. Stock solutions of all drugs were stored at − 20 °C.

### Adenoviral infection

Recombinant adenoviral vector carrying the human mRFP-GFP-LC3 gene was purchased fom HanBio (Wuhan, China). Cells were plated in 12-well plates at a density of 1 × 10^4^ cells per well, and infected at a MOI of 2 with mRFP-GFP- LC3 gene for 24 h. After washing with PBS twice, cells were treated with NEO212 for another 48 respectively.

### Detection of apoptotic cells

Apoptosis was evaluated by using the Annexin V-FITC Apoptosis Detection Kit (BD Biosciences Pharmingen, San Diego, USA) according to the description provided by the manufacturer. After drug treatment, the cells were trypsinized, collected, washed once with PBS and stained with FITC-Annexin V & Propidum Iodide (PI) for 15 min in the dark. The stained cell population were determined using a FACS Calibur instrument (Becton Dickinson, Bedford, MA, USA) and the data were analyzed using FlowJo Software 7.6 (Treestar, Inc., San Carlos, CA). In some experiments, a pan-caspase inhibitor Z-VAD-FMK (20 μM, Z-VAD, KeyGEN BioTECH), and a specific caspase-9 peptide inhibitor Ac-LEHD-FMK (10 μM, Ac-LEHD, KeyGEN BioTECH), were employed along with or without NEO212.

### Cell cycle analysis

Cells growing in 6-well plates were treated by above agents, cells were collected and washed once with PBS, and then re-suspended and fixed in 70% ethanol overnight. After incubation in 1 ml of propidium iodide staining solution (0.1% Triton X-100, 200 μg/ml DNase-free RNase A, 20 μg/ml propidium iodide) for 1 h at room temperature, DNA content was evaluated by a FACS Calibur instrument (Becton Dickinson, USA) and the distribution of cell cycle phases was determined using ModiFit software (Topsham, ME, USA).

### Clone formation assay

Depending on the cell line, 100–350 cells were seeded into each well of a 6-well plate and exposed to 100 μM TMZ, POH, TMZ + POH and NEO212 or DMSO for 48 h, respectively, then drugs were withdrawn and cells grew in normal culture for 14 days, DMSO acted as the control, and subjected to cell colony formation assay. After fixation with acetic acid–methanol (1:4) and staining with diluted crystal violet (1:30), colonies that consisted of > 50 cells were counted and calculated. The colony formation efficiency was calculated with the following formula: Survival Fraction = Clones/Cell numbers × 100%. Three independent experiments were carried out.

### Transmission electron microscope (TEM)

Cells were fixed in TEM stationary solution (2.5% glutaraldehyde in 0.2 M HEPES, G1102, Servicebio Technology) at 4 °C for 4 h, rinsed in PBS, and then embedded in 4% agarose. After fixation in 1% osmium tetroxide for 2 h, the specimens were dehydrated using alcohol and embedded in polybed 812 resin (90529–77-4, SPI). After polymerization at 60 °C for 48 h, ultrathin sections were prepared with the Leica Ultracutuct slicer (Leica EM UC6, Germany), stained with uranyl acetate and lead citrate, and analyzed using TEM (HT7700, HITACHI). Count, measure and analysis on TEM picture were carried out using Fiji ImageJ software.

### Analysis of mitochondrial transmembrane potential (Δψm)

Cancer cells grown in six-well plates overnight were exposed to indicated drugs, then stained with JC-1 (Beyotime, Beijing, China) to demonstrate the state of mitochondrial transmembrane potential according to the manufacturer’s protocol. Briefly, cells were harvested and transferred to 1.5 ml tubes, and then incubated with JC-1 (5 μg/ml) in a 37 °C incubator for 20 min after washing twice with PBS. Subsequently, cells were collected and subjected to flow cytometry (Becton Dickinson) to detect the change of JC-1 florescence. The data were analyzed using FlowJo Software 7.6 (Treestar).

### Cytoplasmic and nuclear extraction

pEnter vector carrying Flag tagged ORF of TFEB (Flag-TFEB) was purchased from Vigene Bioscience (Shandong, China). A2780 and SK-O-V3 cells were seeded into a 6-well plate, incubated overnight and then transfected with pEnter Flag-TFEB using Lipofectamine 3000 (Thermofisher) according to the manufacturer’s instructions to selectively upregulate Flag-TFEB. After 24 h, cells were treated with or without NEO212 in normal medium for 32 h plus EBSS for another16 h, then were subjected to cytoplasmic and nuclear extraction.

Cytoplasmic and nuclear extraction was performed using *MINUTE™ CYTOPLASMIC AND NUCLEAR KIT* (Invent, Beijing, China) according to the manufacturer’s instructions. Briefly, cytoplasmic extraction buffer was added to cells after twice washing with cold PBS and lysed on ice for 5 min; then lysate was centrifuged; supernatant was transferred (cytosol fraction) to a fresh pre-chilled 1.5 ml tube, whereas pellet was added with nuclear extraction buffer, vortexed vigorously and then transferred to a pre-chilled filter cartridge (nuclear fraction). Pre-chilled filter cartridges were centrifuged at 16000 g for 30 s. The filter cartridges were discarded, and protein was collected.

### Immunofluorescence (IF) staining

For immunostaining, cells were fixed with 4% paraformaldehyde and permeabilized with 0.1% Triton X-100 for 15 min. After incubation for 1 h with the following primary antibodies: anti-LC3B (1:300, Cell Signaling Technology, Danvers, MA, USA), anti-TFEB (1:300, Proteintech, PTG, China) and washing with PBS, cells were incubated for 1 h with Alexa 488-conjugated (1:1000) (1:500, Abcam, Shanghai, China) secondary antibodies, washed with PBS. Nuclei were stained by 4′, 6-diamidino-2-phenylindole (DAPI) (Beyotime) for 3 min. Microscopy was done on a confocal laser microscopy (LSM800, Carle Zeiss, Germany).

For quantification of the number of autophagosomes, at least five cells were randomly chosen, all eligible puncta were recorded and analyzed using Fiji ImageJ software [21]. Quantification of GFP and mRFP fluorescence intensity, and colocalization between two different signals were recorded and analyzed using Fiji ImageJ software.

### Fluorescence probe detection

Cells following the above treatment were loaded fluorescence probe Mito-Tracker Green (MTG, Beyotime) for 20 min. After washing 3 times with PBS, the florescence intensities were observed under a confocal laser microscopy (LSM800).

### Western blots

Cells were lysed in Cell Lysis Buffer (20 mM Tris pH 7.5, 150 mM NaCl, 1% Triton X-100) (Beyotime) supplemented with 0.5 mM phenylmethanesulfonyl fluoride (PMSF, Beyotime), and the total cellular protein concentration was determined with a BCA Protein Assay Kit (Thermofisher). 50 μg quantity of protein was separated on SDS-PAGE and transferred onto PVDF membranes (Millipore, Billerica, MA, USA). Membranes were then blocked with 5% evaporated skimmed milk (Bio-rad, USA) in Tris-buffered saline (50 mM Tris-HCl, pH 7.5, 150 mM NaCl) containing 0.1% Tween-20 for 1 h, and probed overnight at 4 °C with the following primary antibodies: antibodies against human LC3B, BECN1, SQSTM1, pho-ATM, γ-H2AX, pho-CHEK1/2, Cyto C, BAX, cl-CASP3, CASP3, cl-CASP9, AKT, pho-AKT, pho-ERK (1:1000; CST), EEA1, LAMP1, LAMP2, TFEB, Lamin B1, GAPDH, ACTB (1:1000; PTG) followed by incubation with horseradish peroxidase coupled secondary anti-mouse or anti-rabbit antibodies (1:2000; PTG) for 1 h at room temperature. The protein bands were visualized using ECL blotting detection reagents (Bio-rad, USA), and developed and fixed onto x-ray films. ACTB or GAPDH or Lamin B1 was served as a loading control dependently.

### In vivo studies

BALB/c nude mice (4–6 weeks of age, female) were purchased from Beijing HFK Bioscience Co., Ltd. (Beijing, China). Mice were housed and handled in laminar flow cabinets under specific pathogen-free conditions according to institutional guidelines and experimental procedures approved by the Institutional Animal Care and Use Committee of Shandong Cancer Hospital and Institute with full respect to the EU Directive 2010/63/EU for animal experimentation. Approximately 5 × 10^6^ SK-O-V3 cells in 100 μl PBS were innoculated s.c. into the left flank of nude mice. When reached approximately 100 mm^3^ in size 1 week later, mice were randomly divided into five groups, and treated once a day for 15 days as follows: DMSO, TMZ, POH, TMZ + POH and NEO212. Size of local tumors and mouse body weight were calculated by measuring two perpendicular diameters (length and width) every two days using a caliper, and the volume was calculated according to the formula: tumor volume (mm^3^) = 1/2 × (length × square width). The mice were sacrificed after completion of treatment and the tumors were separated and weighted.

### Immunohistochemical (IHC) staining

The xenograft tumor tissues were fixed and paraffin-embedded for section. After routinely dewaxing and hydration, antigen in specimens proceeded to be repaired, the slides were blocked for endogenous peroxidase activity, preincubated with goat serum, and then stained with anti-cl-CASP3 (Servicebio Technology)) for 1 h at room temperature. Secondary staining was carried out with HRP- conjugated anti-rabbit IgG and DAB peroxidase substrate.

### Hematoxylin-eosin (H&E) staining

The slides were deparaffinized then stained with hematoxylin (2 min), rinsed with distilled water, rinsed with 0.1% hydrochloric acid in 50% ethanol, rinsed with tap water for 15 min, stained with eosin for 1 min, and rinsed again with distilled water. The slides were then dehydrated and mounted with coverslips.

### Statistical analysis

Statistical significance was evaluated with data from at least two independent experiments or at least five duplicates. GraphPad Prism 6.02 (GraphPad Software, San Diego, CA, USA) was used for data analysis. Statistical analysis was carried out using Student t-test for two groups as well as one-way ANNOVA for more than two groups. Data are presented as the mean ± SD. For all statistical tests, significance was established at *P* < 0.05.

## Results

### NEO212 exerts stronger cytotoxicity than its individual constituents in vitro

**Fig. 1:**
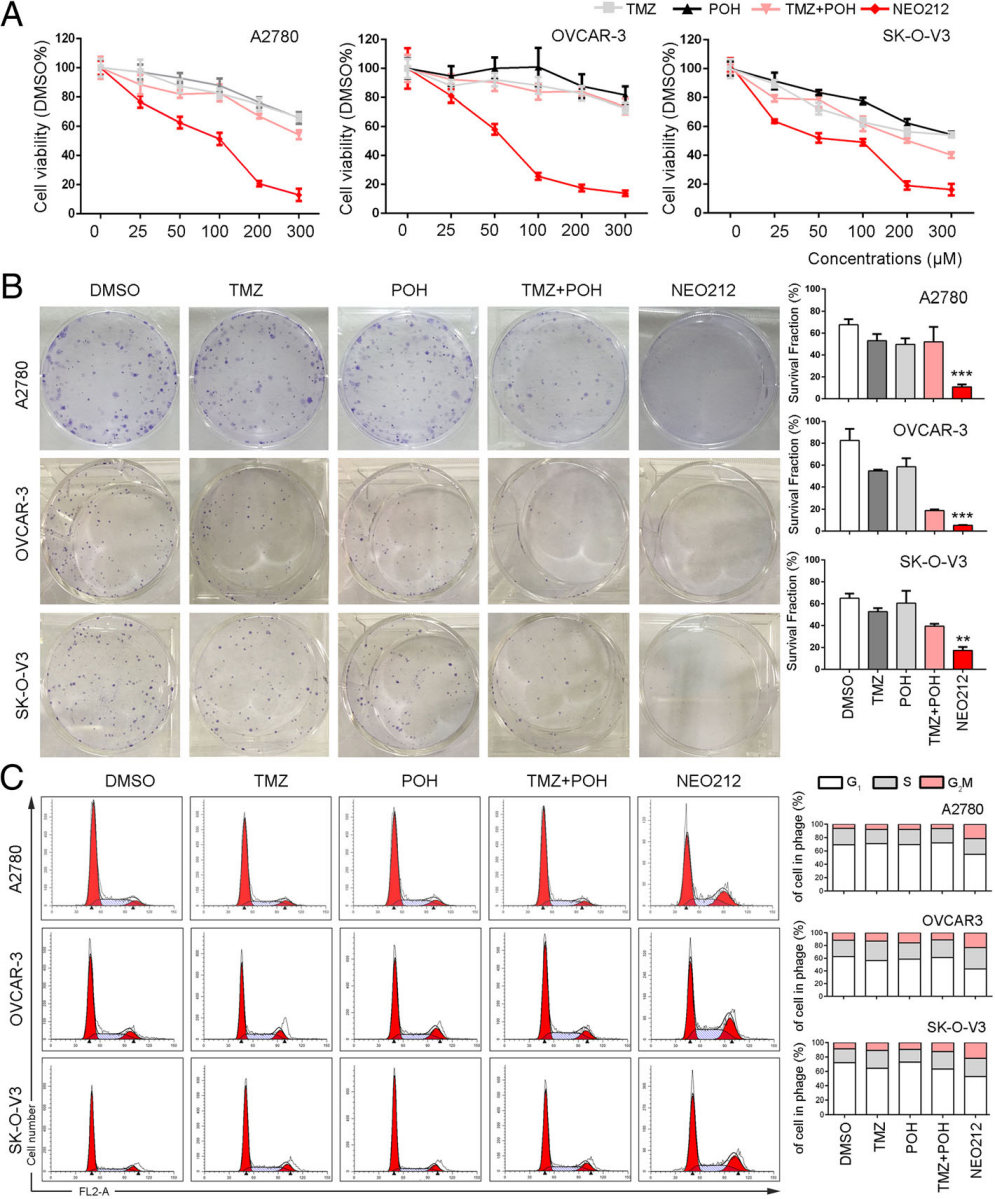
NEO212 exerts stronger cytotoxicity than its individual constituents in vitro. **a** A2780, SK-O-V3 and OVCAR-3 cells were treated with 0, 25, 50, 100, 150, 200, 300 μM TMZ, POH, TMZ + POH and NEO212 respectively for 48 h, DMSO acted as the control, and then subjected to MTT assay. Absorbance value was calculated and standardized to DMSO group. Three independent experiments were performed. **b** The above cells were treated with 100 μM TMZ, POH, TMZ + POH and NEO212 or DMSO for 48 h, respectively, then drugs were withdrawn and cells grew in normal culture for 14 days, DMSO acted as the control, and subjected to cell colony formation assay Statistical differences of cell colony formation assay were calculated and presented as mean ± SD. **c** A2780, SK-O-V3 and OVCAR-3 cells were treated with 100 μM TMZ, POH, TMZ + POH, NEO212 or DMSO respectively for 48 h, and the cell cycle distributions were analyzed. The results shown are means ± SD; *p < 0.05; **p < 0.01; ***p < 0.001

To clarify the cytotoxic potency of NEO212 against ovarian cancer, three ovarian cancer cell lines A2780, SK-O-V3 and OVCAR-3 were employed and exposed to treatment with control (Dimethyl Sulphoxide, DMSO), TMZ, POH, TMZ plus POH combination (TMZ + POH) and NEO212, respectively. Cell viability detected by MTT assay was shown in Fig. 1a. NEO212 inhibited cell viability in a dose-dependent manner compared to its individual constituents and their combination in all three cancer cell lines, indicating the inhibitory effect of NEO212 on tumor cell proliferation independent of cell type.

In addition, a colony formation assay was also carried out, as shown in Fig. 1b, colony formation was suppressed by NEO212 more potently than its constituents and their combination. These data support the inhibitory role of NEO212 in ovarian cancer cell growth and colony formation.

It has been reported TMZ has the ability to alkylate DNA, and leads to DNA double strand breaks (DSBs) and DNA damage response (DDR) [22], which transmitted DNA damage signal to regulate cell cycle for DNA damage repair, thus the effect of NEO212 on cell cycle was also analyzed. As shown in Fig. 1c, NEO212 increased the population at the G_2_M phase and decreased the population at the G_1_ in all three ovarian cancer cell lines, indicating NEO212 mediated G_2_M arrest.

### NEO212 inhibits ovarian cancer proliferation in vivo

**Fig. 2:**
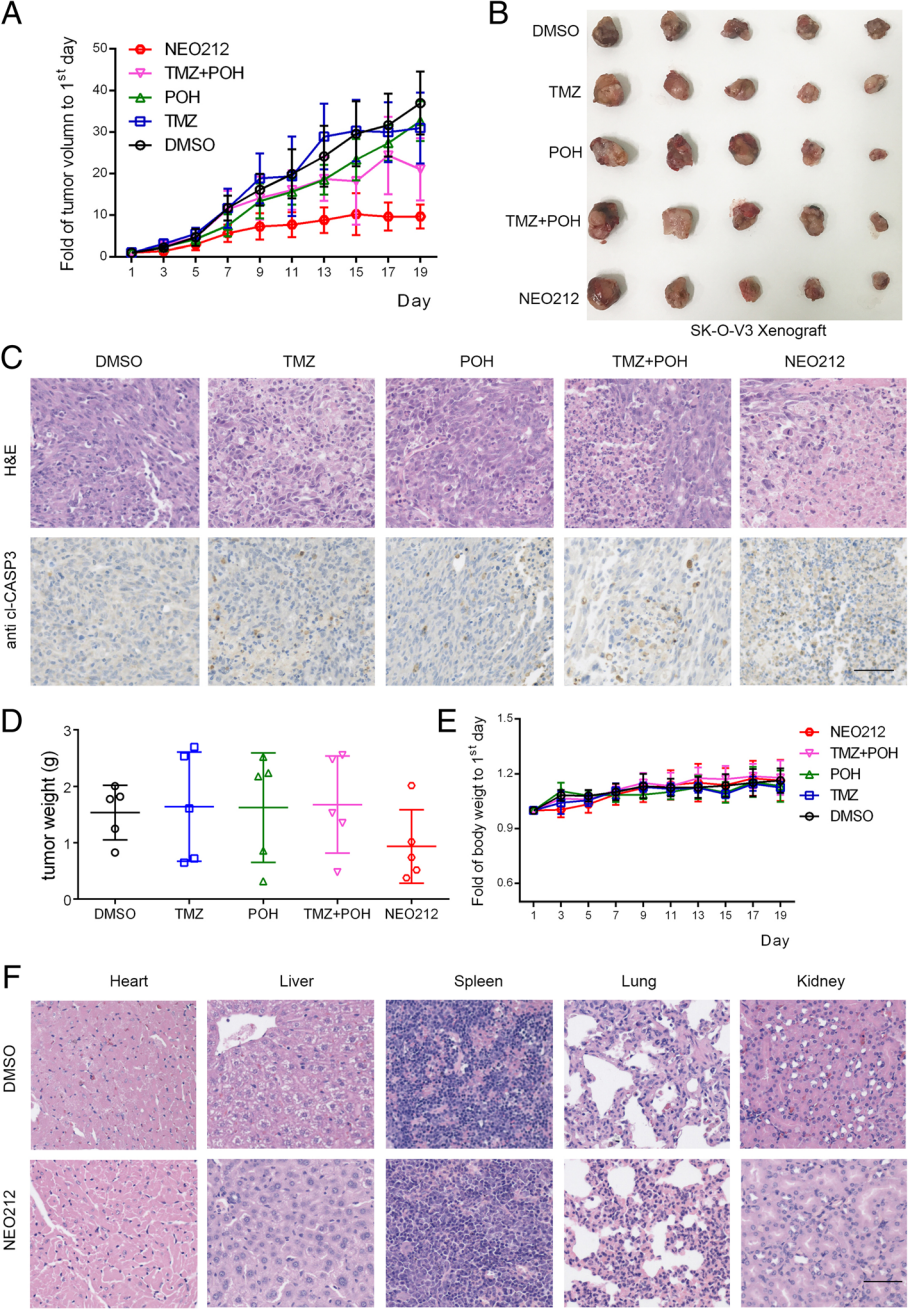
NEO212 inhibited ovarian cancer proliferation in vivo. SK-O-V3 xenograft tumor was established and treated as follows: DMSO, TMZ (25 mg/kg), POH (25 mg/kg), POH (11 mg/kg) mixed with TMZ (14 mg/kg) (mimicking the dosage of the individual components contained in 25 mg/kg TMZ − POH), NEO212 (25 mg/kg). Tumor growth curves (**a**), tumor image (**b**) of treated tumors with different treatment were detected. After tumor dissection, tumors were subjected to H&E staining and IHC staining via anti cl-CASP3 (**c**), tumor weight (**d**). Mice body weight (**e**), major organs H&E staining (**f**) were also evaluated. Scale bars: 50 μm

The inhibitory effect of NEO212 on the inhibition of NSCLC proliferation in vivo was also tested. Murine xenograft tumors were established and treated as described in “*Materials and Methods*”. As expected, tumors administrated with NEO212 grew slower than those with other chemicals (Fig. 2a and b), indicating that the therapeutic efficacy of NEO212 was substantially stronger than that of TMZ, POH, or their combination. After tumor dissection, tumors were subjected to H&E staining. The results demonstrated necrotic foci were observed in NEO212 treated group but not in others (Fig. 2c, upper). NEO212 induced apoptosis was also detected by IHC staining via anti-cleaved CASPAPSE 3 (cl-CASP 3). As shown in Fig. 2c, lower, NEO212 treatment led to stronger cl-CASP3 staining than its individual constituents and their combination, indicating NEO212 was capable to induce apoptosis. No statistical differences but a diminished tendency of separated tumors’ weight were observed in NEO212 treated group, which might ascribe to different initial tumor size and small- number of samples (Fig. 2d).

We also evaluated the potential side effect caused by NEO212. No statistically significant differences in the body weights were observed between the mice in NEO212-treated group and mice in control group (Fig. 2e). Meanwhile, NEO212 administration also failed to cause morphological and histological changes of major organs in nude mice, indicating NEO212 functioned on tumor cells selectively but not caused the damage for the normal tissue (Fig. 2f). Taken together, the present study provides further insights into the cytotoxic effect of NEO212 against ovarian cancer in vitro and in vivo.

### NEO212 induces DNA damage and mitochondrial apoptosis

**Fig. 3:**
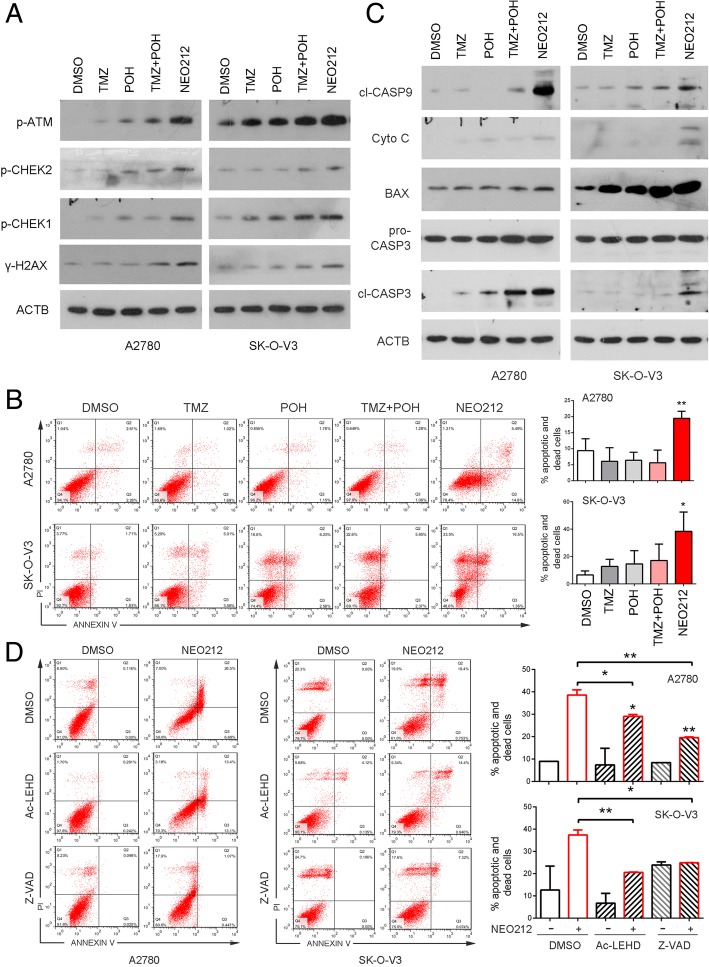
NEO212 induces DNA damage and mitochondrial apoptosis. **a-c** Cells were treated with 100 μM TMZ, POH, TMZ + POH, NEO212 or DMSO respectively for 48 h. **a** Western blot analysis demonstrated p-ATM, p-CHEK1, p-CHEK2, p-H2AFX and ACTB expression in above drug-treated A2780 and SK-O-V3 cells. **b** Cells were subjected to apoptosis assay using Annexin-V &PI staining. **c** Lysates from above drug-treated A2780 and SK-O-V3 cells were subjected to western blot using following antibodies against cleaved CASP9, pro-CASP3, cleaved CASP3, Cytochrome C (Cyto C), BAX and ACTB. **d** A2780 and SK-O-V3 cells were treated with 100 μM NEO212 for 48 h with or without Ac-LEHD or Z-VAD, and then apoptosis assay using Annexin-V &PI staining. The results shown are means ± SD; *p < 0.05; **p < 0.01

As described above TMZ induced DSBs trigger the recruitment of Ataxia telangiectasia mutated (ATM), a serine/threonine protein kinase, to the damaged site, which in turn phosphorylates H2A histone family member X(H2AFX) resulting in foci formation at the damage side [23] and transmits the DNA damage signal to downstream substrates checkpoint kinases 1 and 2 (CHEK1/CHEK2) [24], thus we detected the DNA damage related protein expression when A2780 and SK-O-V3 cells suffering NEO212 treatment using western blot. As shown in Fig. 3a, NEO212 induced higher level of phosphorylation of ATM at the Ser1981 site, and CHEK1 and CHEK2 at the Ser345 and Thr68 site respectively, as well as that of H2AFX at the Ser139 site compared with its individual constituents and their combination, suggesting that NEO212 can lead to a stronger DNA damage in ovarian cancer cells.

Given that DNA damage can trigger apoptosis, the effect of NEO212 on cell apoptosis was also detected. As shown in Fig. 3b and Additional file 1: Figure S1A, flow cytometry analysis displayed much more apoptotic and dead cells when treated with NEO212 other than treated with its individual constituents and their combination in all three ovarian cancer cells. Studies have shown that two signal pathways participate in cell apoptosis, one is the death receptor pathway and the other is the mitochondrial pathway. To verify which one involved in NEO212 induced apoptosis, apoptosis-related gene expression was detected in A2780 and SK-O-V3 cells. As shown in Fig. 3c, NEO212 treatment led to an increase in expression of cleaved Caspase3 (cl-CASP3) but no influence on pro-Caspase3 (pro-CASP3). Importantly, BAX, Cytochrome C (Cyto C) and cleaved Caspase 9 (cl-CASP9), vital markers in mitochondrial pathways, were also elevated significantly in response to NEO212 treatment, indicating that mitochondrial apoptosis is activated and plays a key role in NEO212 mediated cell death.

To further confirm the forms of NEO212-induced cell death, Z-VAD-FMK (Z-VAD), a pan-caspase inhibitor, and Ac-LEHD-FMK (Ac-LEHD), a specific caspase-9 peptide inhibitor, were introduced to apoptosis analysis in afore-mentioned cells along with NEO212. As shown in Fig. 3d, both pan-inhibitor and caspase-9 inhibitor significantly decreased NEO212 induced cell apoptosis. These results further suggest that NEO212 caused apoptosis via the caspase-dependent mitochondrial pathway. However, caspase inhibitors cannot completely block NEO212 induced apoptosis; therefore, there may also be a caspase-independent pathway involved in it-induced apoptotic process (Fig. 3d).

### NEO212 promotes mitochondrial dysfunction

**Fig. 4:**
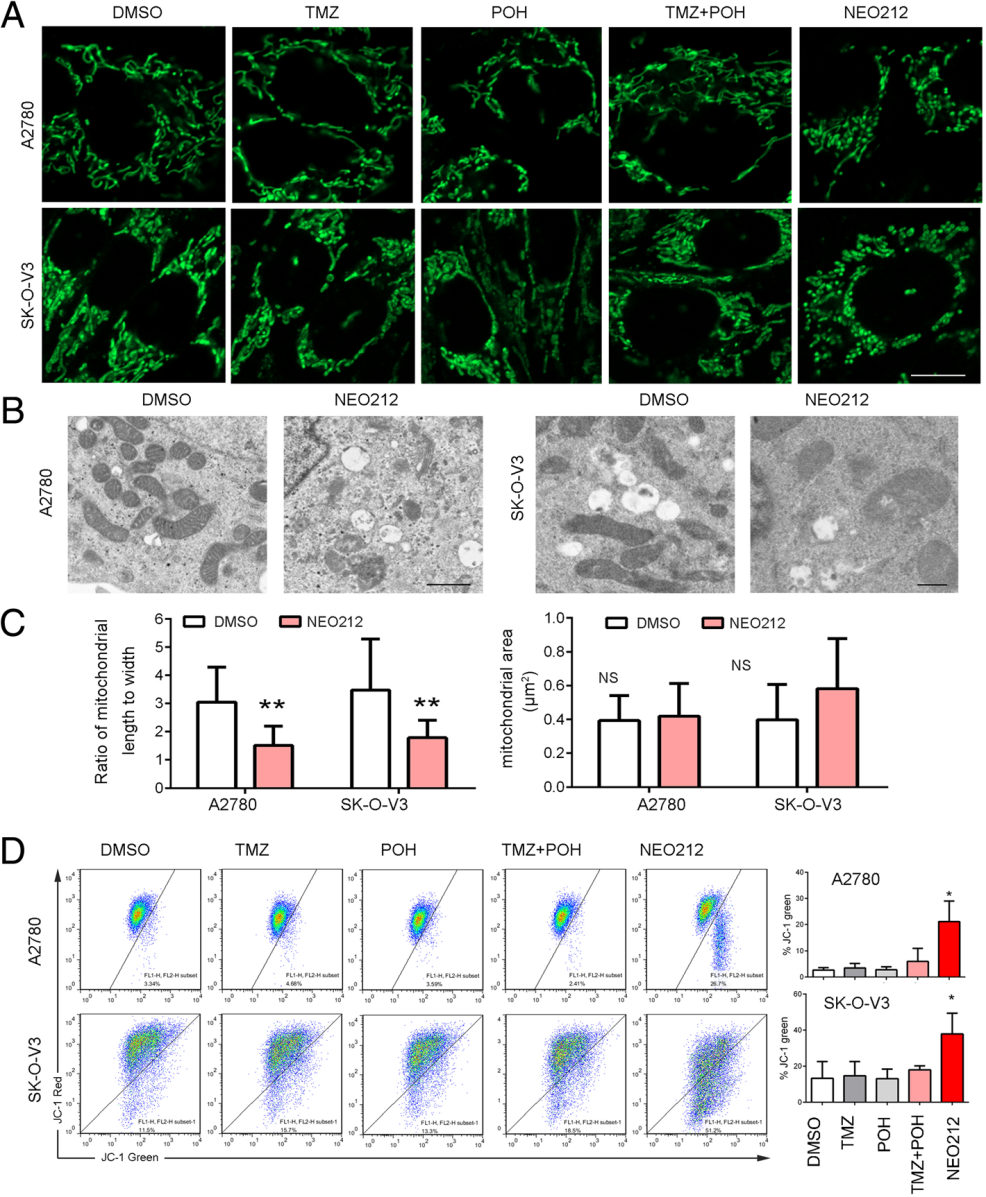
NEO212 promotes mitochondrial dysfunction. **a** A2780 and SK-O-V3 cells treated with 100 μM TMZ, POH, TMZ + POH, NEO212 or DMSO for 48 h respectively were stained with MTG. Scale bars: 10 μm. **b-c** Mitochondria structure in A2780 and SK-O-V3 cells treated with 100 μM NEO212 or DMSO was observed by TEM. Scale bars: 0.5 μm. Mitochondrial shape and area were measured using Fiji Image J. **d** A2780 and SK-O-V3 cells were treated with 100 μM TMZ, POH, TMZ + POH, NEO212 or DMSO for 48 h respectively, and detected using JC-1 flow cytometry. The results shown are means ± SD; *p < 0.05; **p < 0.01; ***p < 0.001, NS = no significance

Mitochondrial fusion and fission occur in a balanced frequency and thus relatively constant tubular morphology is maintained, but this subtle balance can be deteriorated when treated with chemical agents. Hence the next step aimed to check the effect of NEO212 on mitochondrial fusion and fission in ovarian cancer cells. First, we used the mitochondrial fluorescence probe Mito-Tracker Green (MTG) to mark and observed the mitochondria using a confocal microscopy. As shown in Fig. 4a, NEO212 induced accumulation of fragmented mitochondria with shorter lengths and fewer numbers of branches due to a lack of mitochondrial fusion, whereas other drugs induced that of tubular mitochondria in both A2780 and SK-O-V3 cells. Next, NEO212 treated cells were subjected to transmission electron microscope (TEM) observation, which demonstrated that the morphology of mitochondria became more round and shorter, and the overall shapes of the mitochondria were grossly distorted and the inner mitochondrial matrices were either severely damaged or almost absent compared to the control groups in above mentioned three cell lines although their sizes seemed unchanged (Fig. 4b, c and Additional file 1: Figure S1B), indicating NEO212 disturbs mitochondrial morphology and promotes its fission.

It has been reported that the ability of mitochondrial membranes to fuse is regulated by their potential, dissipation of transmembrane potential inhibits mitochondrial fusion and promotes its fission [25], thus the mitochondrial transmembrane potential (ΔΨm) was evaluated by JC-1 probe (5,5′,6,6′-Tetrachloro-1,1′,3,3′-tetraethyl-imidacarbocyanine iodide) in our study. As shown in Fig. 4d, NEO212 led to a significant decrease in ΔΨm compared to other drugs, as indicated by increased JC-1 fluorescent intensity of green to red in A2780 and SK-O-V3. Cells. These observations support that NEO212 promotes mitochondrial fission, and damages mitochondrial function.

### Relationship of NEO212 induced apoptosis and autophagosome accumulation

**Fig. 5:**
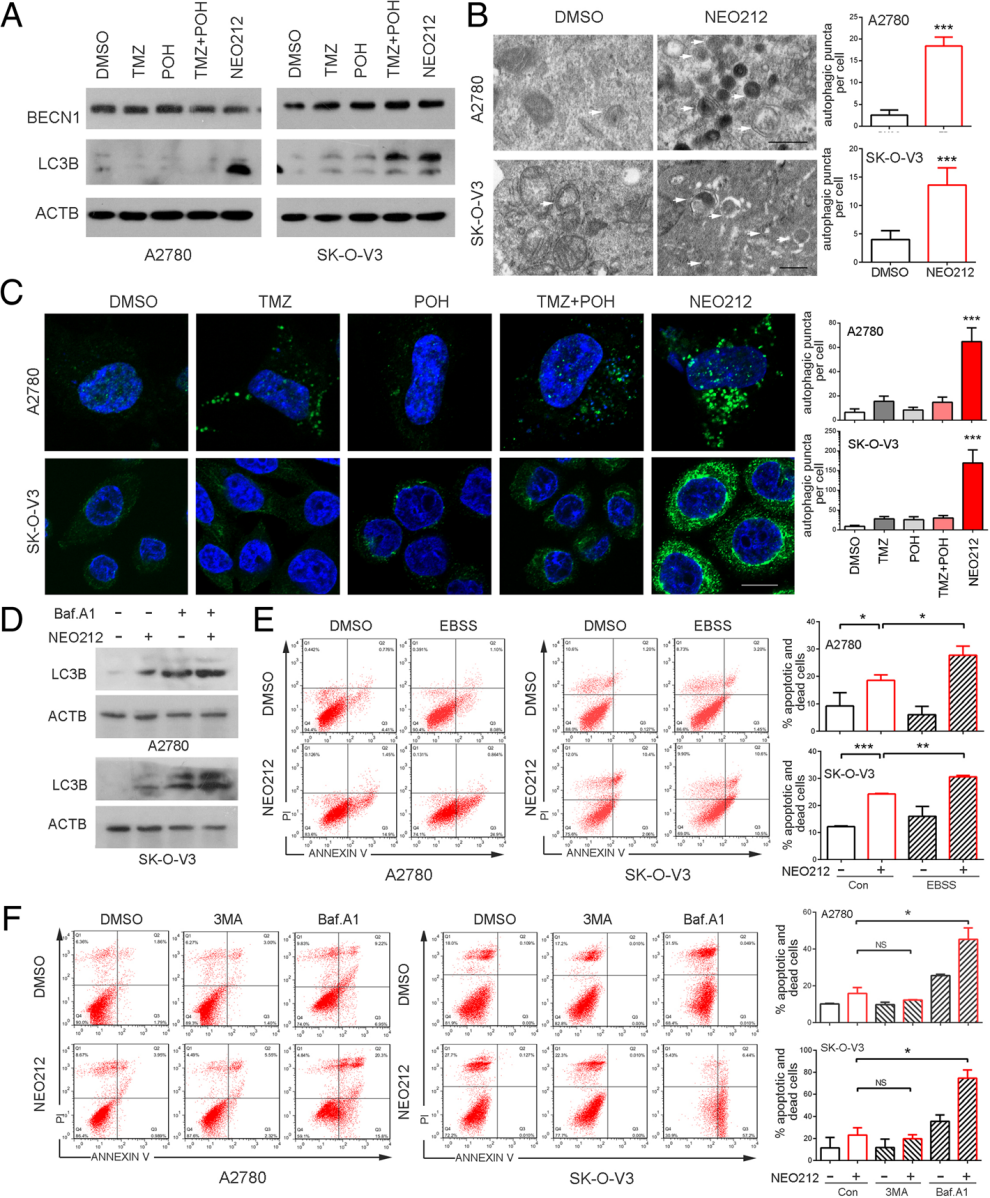
Relationship of NEO212 induced autophagosome accumulation and apoptosis. **a** Cells were treated with 100 μM TMZ, POH, TMZ + POH, NEO212 or DMSO respectively for 48 h in A2780 and SK-O-V3 cells. Western blot analysis demonstrated BECN1, LC3B and ACTB expression; (**b**) Autophagic vacuoles in A2780 and SK-O-V3 cells treated with 100 μM NEO212 or DMSO were observed by transmission electron microscopy (TEM). The arrow indicates autophagic vacuoles. Scale bars: 0.5 μm. **c** The above drug-treated cells were inspected under confocal laser microscopy to detect LC3B puncta by immunofluorescence. Scale bars: 10 μm. Number of autophagic vacuoles was calculated using Fiji Image J software. **d** A2780 and SK-O-V3 cells treated with 100 μM NEO212 or DMSO were subjected to western blot to detected LC3B and ACTB expression in the presence or absence of Baf.A1. **e-f** A2780 and SK-O-V3 cells were treated with 100 μM NEO212 for 48 h with or without EBSS (**e**) or 3 M, Baf.A1 (**f**), and then apoptosis assay using Annexin-V &PI staining. The results shown are means ± SD; *p < 0.05; **p < 0.01; ***p < 0.001, NS = no significance

Studies have suggested that autophagy can break down the damaged organelles to provide energy to maintain cell homeostasis in response to some stress response in cancer cells [26]. Therefore, we first studied the effect of NEO212 on autophagy of ovarian cancer cells. As shown in Fig. 5a, autophagy was activated significantly when treated by NEO212 compared to its individual constituents or their combination in A2780 and SK-O-V3 cell lines, as evidence from the increases in the amount of LC3B-II. However, NEO212 seemed to have no obvious effects on the expression of BECN1 in A2780 and SK-O-V3 cells, indicating it didn’t directly depend on the activation of BECN1 pathway to induce autophagy. This phenomenon was confirmed by TEM, as shown in Fig. 5b, NEO212 treatment significantly increased intracellular autophagic vacuoles shown as double membrane vesicles with visible cytoplasm contents. Furthermore, we checked the formation of autophagosomes by staining endogenous LC3B. We found that NEO212 treatment increased intracellular autophagosomes compared to its individual constituents and their combination, as demonstrated by an accumulation of LC3B-positive spot-like structures in NEO212 treated A2780 and SK-O-V3 cell lines (Fig. 5c). Therefore, our data suggest NEO212 induces autophagosome accumulation.

To rule out the possibility that NEO212 promoted excessive autophagic degradation which led to the failure in autophagosome accumulation, we treated cells combined with Bafilomycin A1 (Baf.A1). As shown in Fig. 5d, Baf.A1-elevated LC3B-II expression arrived at a similar level in cells with or without presence of NEO212, while NEO212 alone induced an increase in LC3B-II expression, indicating a promotion of excessive autophagic degradation was not involved in the process that NEO212 functioned.

Multiple papers have been published that have identified and/or characterized the cytoprotective function of autophagy, primarily in tumor cells exposed to chemotherapy or radiation [27]. In order to illuminate the role of autophagy in NEO212 induced apoptosis, Earle’s Balanced Salt Solution (EBSS) was employed for autophagy induction along with or without NEO212. As shown in Fig. 5e, autophagy introduction by EBSS, instead of the protective role, augmented NEO212 induced apoptosis in A2780 and SK-O-V3 cells. Previous studies have established the autophagy inhibitor 3MA and Baf.A1 played quite opposite role in TMZ’s cytotoxicity [16]. Thereby, both 3MA and Baf.A1 were employed to cooperate with NEO212. As show in Fig. 5f, 3MA with the capability to prevent autophagy at an early stage, appeared no any positive or negative effect on NEO212 induced apoptosis. Nevertheless, Baf.A1 that prevents autophagy at a late stage sensitized tumor cells to NEO212 by inducing apoptosis significantly, indicatingNEO212 might interrupt autophagy flux, although it induced autophagosome accumulation.

### NEO212 blocks autophagy flux and TFEB nuclear translocation

**Fig. 6:**
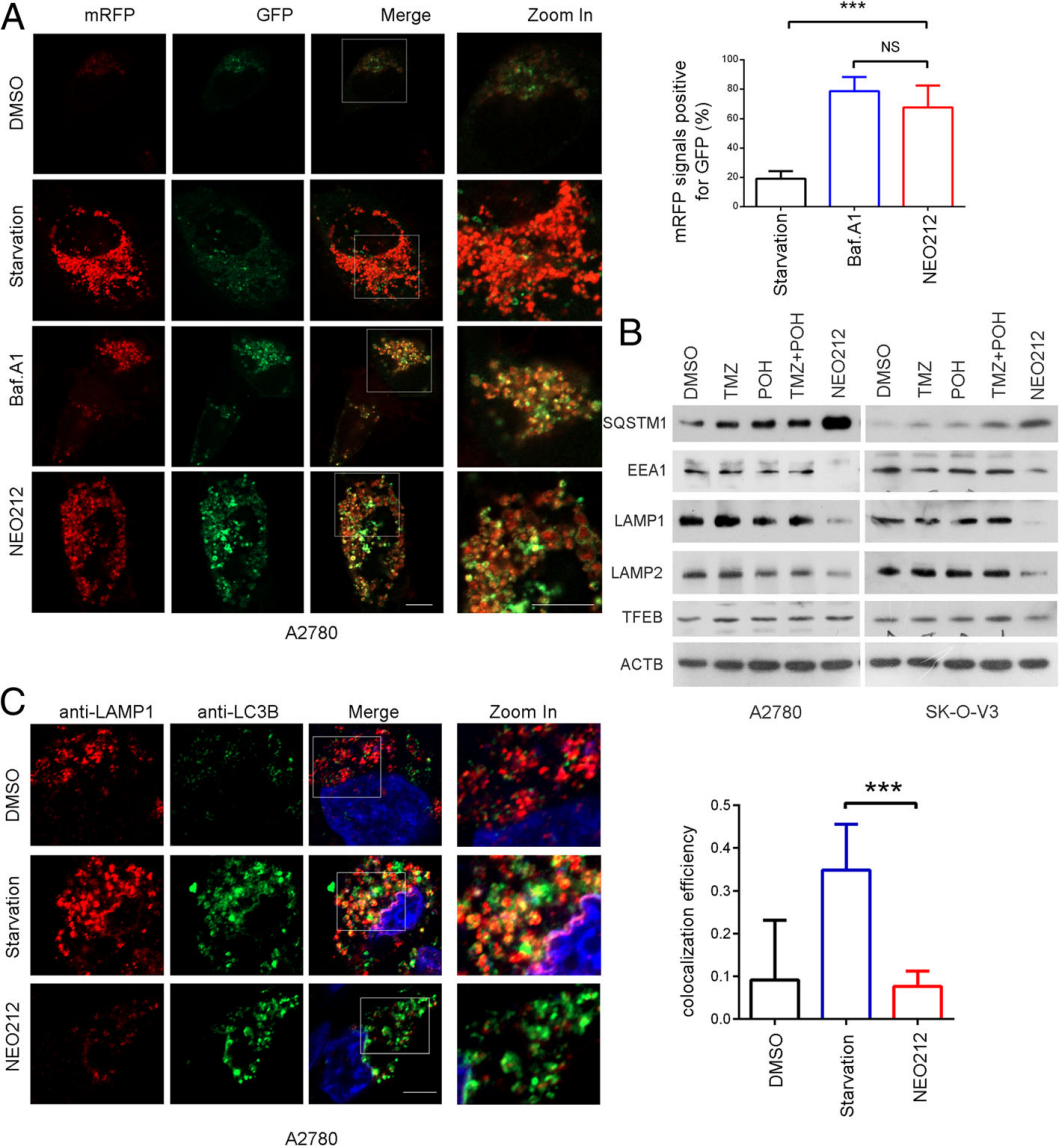
NEO212 blocks autophagy flux. **a** A2780 cells expressing mRFP-GFP-LC3 were starved or treated with 1 nm Baf.A1 or 100 μM NEO212 for 48 h and imaged by confocal microscopy. Scale bars: 10 μm. Statistical analysis of the percentage of puncta mRFP signals that were positive for GFP using Fiji Image J. **b** Cells were treated with 100 μM TMZ, POH, TMZ + POH, NEO212 or DMSO respectively for 48 h in A2780 and SK-O-V3 cells. Western blot analysis demonstrated SQSTM1, EEA1, LAMP1, LAMP2, TFEB and ACTB expression. **c** A2780 cells were either starved or treated with NEO212, and stained with anti-LAMP1 and anti-LC3B antibodies and imaged by confocal microscopy. Scale bars: 10 μm. Statistical analysis of the colocalization coefficient of LAMP1 and LC3B using Fiji Image J. The results shown are means ± SD; ***p < 0.001, NS = no significance

To confirm the role of NEO212 in autophagy flux, we transfected A2780 cells with the devised fusion protein mRFP-GFP-LC3 via adenovirus vector, which labels autophagosomes yellow because of superposition of GFP and mRFP signals, and autolysosomes red as the low lysosomal pH quenches the GFP signal [28]. As shown in Fig. 6a, in starved cells, most of the puncta lost the GFP signal and retained the mRFP signal. However, in cells treated by NEO212, like Baf.A1 but unlike those starved, quenching of the GFP was significantly diminished as indicated by the retention of both the mRFP and GFP signals.

Furthermore, SQSTM1, a selective autophagic adaptor incorporated with LC3B into autophagosomes and degraded by lysosomal hydrolysis, was significantly elevated by NEO212 treatment, indicating NEO212 plays an inhibitory role in autophagy flux (Fig. 6b). Autophagy flux signified autophagosome transported dysfunctional organelles and other cellular contents into lysosome for degradation and recycling, manifesting the important role of lysosome, thus we detected the expression of lysosomal related proteins including early endosome antigen 1 (EEA1), a marker for early endosomes, lysosome- associated membrane protein (LAMP) 1 and 2, markers for mature lysosome. As shown in Fig. 6b, NEO212 significantly down-regulated of EEA1, LAMP1 and LAMP2 compared to other drugs, indicating NEO212 perturbed lysosomal mature and activity. However, NEO212 failed to affect the expression of TFEB, a transcriptional regulatory factor that can regulate the expression levels of a series of genes related to lysosome.

Furthermore, we investigated autophagosome-lysosome fusion via observing the colocalization of LC3B with LAMP1. As shown in Fig. 6c, starvation led to an overlap of LC3B with LAMP1, whereas NEO212 caused a significant decrease in the coefficient of LC3B-LAMP1 colocalization in A2780 cells. Notably, NEO212 induced LAMP1 down-expression significantly (Fig. 6b), which should not be ruled out as a partial reason for the low level of LC3B-LAMP1 colocalization.

**Fig. 7:**
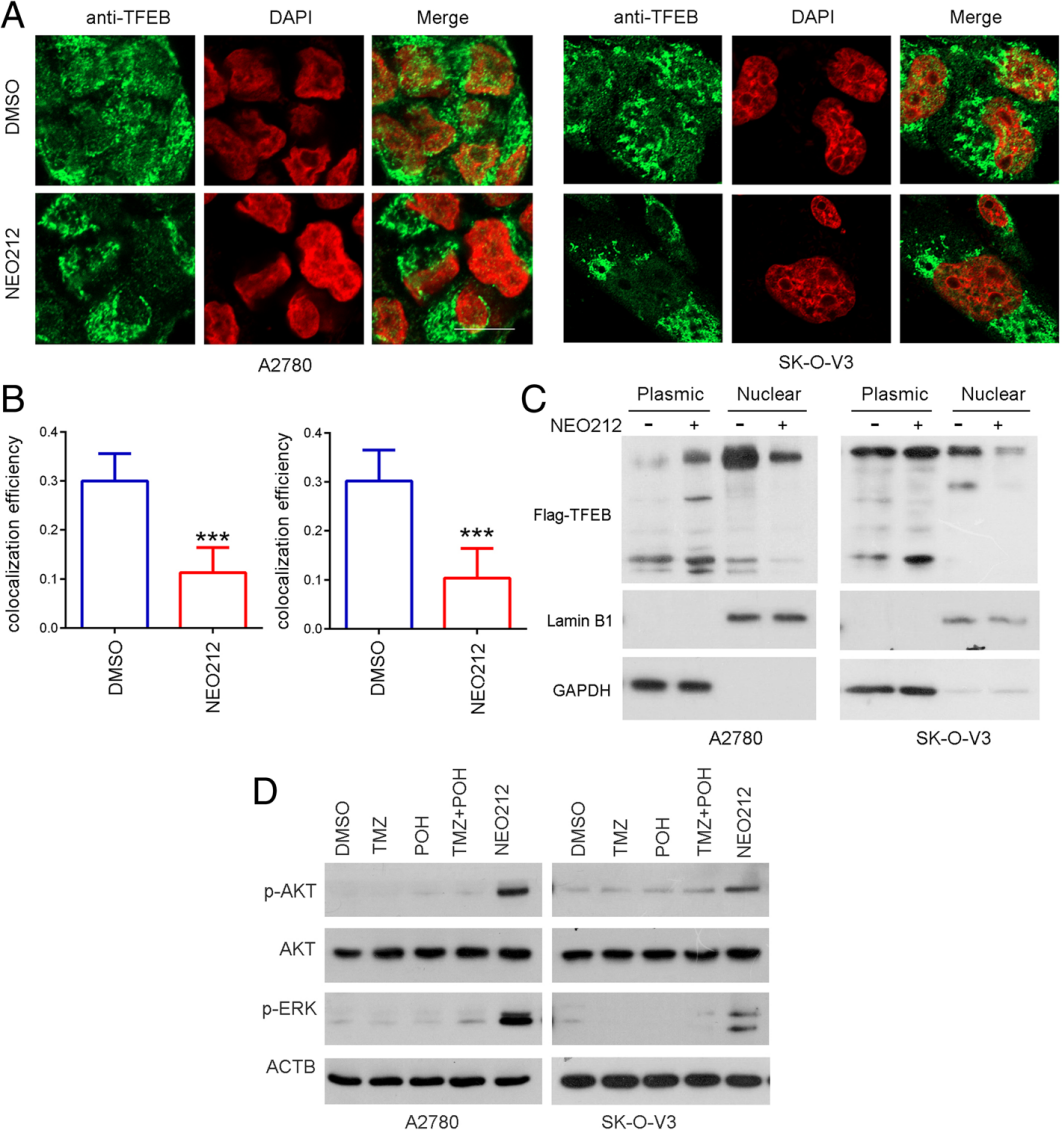
NEO212 blocks TFEB nuclear translocation. **a-b** A2780 and SK-O-V3 cells were treated with 100 μM NEO212 or not and stained with anti-TFEB antibody and imaged by confocal microscopy (**a**). Scale bars: 10 μm. Statistical analysis of the colocalization coefficient of LAMP1 and LC3B using Fiji Image J (**b**). **c** A2780 cells expressing Flag-TFEB were starved in present or absent with 100 μM NEO212 underwent cytoplasmic and nuclear extraction. Then the cytoplasmic and nuclear lysates were subjected to western blot analysis for demonstrating Flag-TFEB expression using anti Flag antibody. GAPDH acts as control for cytoplasmic protein, Lamin B1 acts as control for nuclear protein; (**d**) Cells were treated with 100 μM TMZ, POH, TMZ + POH, NEO212 or DMSO respectively for 48 h in A2780 and SK-O-V3 cells. Western blot analysis demonstrated p-AKT, AKT, p-ERK, and ACTB expression in above drug treated cells. The results shown are means ±SD, ***p < 0.001

Previous studies have reported that TFEB positively regulates the transcription of genes involved in all steps of lysosome biogenesis. It promotes lysosomal proliferation, acidification, and exocytosis [29]. Since the initial characterization of TFEB as a transcriptional modulator of the lysosomal system, cytoplasm-to-nucleus translocation of TFEB has been identified as the main mechanism underlying regulation of TFEB activity [30]. Therefore, we studied the effect of NEO212 on TFEB nuclear translocation although it failed to alert TFEB expression. As shown in Fig. 7a and b, TFEB was transferred into the nucleus after EBSS stimulation for 16 h, which was significantly inhibited by NEO212 treatment for 48 h, indicating that NEO212 was capable to block the nuclear translocation of TFEB. Moreover, Flag tagged-TFEB vector was enrolled and subjected to transfection into A2780 and SK-O-V3 cells, TFEB expression was detected in cytoplasm and nucleus, respectively. Consistently, as shown in Fig. 7c, NEO212 induced up-regulation of TFEB in cytoplasm but downregulation in nucleus, indicating its capability to block the nuclear translocation of TFEB.

In addition, nuclear localization and activity of TFEB is regulated by serine phosphorylation mediated by the extracellular signal-regulated kinase (ERK) [30] and serine/threonine kinase AKT [31]. As shown in Fig. 7d, NEO212 was capable to induce high level of phosphorylated ERK and AKT obviously, which further strengthens the evidence NEO212 blocked the nuclear translocation of TFEB, suggesting that NEO212 might induce TFEB phosphorylation through the AKT pathway, and then inhibit its nuclear translocation. Taken together, our results suggest that NEO212 induces lysosomal dysfunction and blocks TFEB nuclear translocation through up-regulating ERK/AKT phosphorylation.

## Discussion

In current study, we provide a novel report on the cytotoxic effect of NEO212 on ovarian cancer in vitro and in vivo, and the underlying mechanisms. TMZ, a novel alkylating agent, had insignificant activity in conspicuous therapeutic effect for ovarian cancer [32], whereas the newly designed compound NEO212, a novel temozolomide analog, exerted stronger cytotoxicity than its individual constituents, as evidence from stronger inhibition of cell proliferation and colony formation, along with higher level of G_2_M arrest, DNA damage, mitochondrial dysfunction and apoptosis, thereby proposing NEO212 as a potential candidate for ovarian cancer therapy.

Here, we believed that mitochondria were one of main sites that NEO212 exerted its cytotoxicity. We found that NEO212 damaged the mitochondrial morphology and activity, as proved by increased fission, destroyed shape and transmembrane potential collapse when treated by NEO212 in ovarian cancer, similar to those in NSCLC [7] and NPC [9] cells. Diversely, in NSCLC and NPC cells, NEO212 induced ROS accumulation was the key contributor to its cytotoxicity because it can be reversed by two ROS scavengers, catalase (CAT) and N-acetyl-L-cysteine (NAC); However, NEO212 seemed not alter the level of intracellular ROS accumulation (data not shown), indicating alternative pathway was involved in. We supposed NEO212 as an alkylating agent might works due to its capability to methylate mitochondrial DNA directly and induce its dysfunction, although this hypothesis needs further investigation.

Importantly, our data supported that NEO212 induced apoptosis via mitochondrial pathway. This is because NEO212 was capable to regulate a series of mitochondrial apoptosis related genes, such as BAX, Cyto C and cl-CASP9. Cyto C, a pro-apoptotic factor, releases on the outer surface of the inner mitochondrial membrane at early steps of apoptosis which can be initiated by the pro-apoptotic protein BAX, combining with some cytosolic proteins, activates conversion of the latent apoptosis-promoting protease pro-CASP9 to its active form, and finally mitochondrial apoptosis. This conclusion was supported by a pan CASP inhibitor and a specific CASP9 inhibitor analysis, both of which succeeded in relieving NEO212 induced apoptosis, indicating a caspase dependent manner involved. Notably, in our previous study, the pan caspase inhibitor failed to prevent cells from NEO212 cytotoxicity in NSCLC cells [7], which might attribute to the cell specificity.

Dysfunctional and damaged mitochondria initiate the protective mechanisms, such as autophagy, a conserved eukaryotic catabolic reaction for removal and recycling, thereby it has been proposed as a mechanism of chemoresistance to alkylating drugs [33]. Depending on the cellular context, autophagy may lead to tumor cell survival or death. Inhibition of autophagy by 3MA failed to increase TMZ sensitivity as observed with Baf.A1 but, instead, enhanced TMZ chemoresistance of glioma cells [16], since TMZ induced an autophagy-associated increase in ATP production, which was blocked by pre-incubation with autophagy inhibitor 3MA, and increased non-apoptotic cell death [34]. In current study, we also explored the association of NEO212 induced autophagosome accumulation and apoptosis. 3MA appeared no any positive or negative effect on NEO212 induced apoptosis, whereas Baf.A1 that prevents autophagy at a late stage sensitized tumor cells to NEO212 by inducing apoptosis, indicating NEO212 might interrupt autophagy flux. Lysosomotropic agents, like Baf. A1 and chloroquine (CQ), impair lysosome function and its fusion with autophagosome and inhibit autophagic flux, and in turn eliminate the protective role of autophagy, which have been used successfully to overcome chemoresistance against alkylating drugs. In current study, we observed that NEO212 quenched GFP-LC3 degradation, down-regulated a series of lysosome related gene expression, and blocked the autophagic flux, although it induced significant accumulation of autophagosomes, implying NEO212 might avoid from autophagy-mediated chemoresistance.

Collectively, we add a novel insight into the role and underlying mechanisms of NEO212 in ovarian cancer. We have demonstrated NEO212 exerted stronger cytotoxicity than its individual constituents, as evidence from stronger inhibition of cell proliferation and colony formation, along with higher level of G_2_M arrest, DNA damage, mitochondrial dysfuncton and apoptosis. Besides, NEO212 was capable to down-regulate a series of lysosome related gene expression via hindering the nuclear translocation of TFEB, and in turn block the autophagic flux, implying NEO212 might as a promising therapeutic agent against ovarian cancer.

## Conclusions

In the current study, we revealed the potential anticancer property of NEO212 in ovarian cancer cells, as evidence from cell proliferation inhibition, G_2_/M arrest, DNA damage, mitochondrial fission and apoptosis. Importantly, we observed that NEO212 blocked autophagy flux which significantly facilitated it induced apoptosis and was largely because NEO212 inhibited the nuclear translocation of transcription factor EB (EB), and impaired the lysosomal function, implying NEO212 might avoid from autophagy mediated chemoresistance, thus proposing NEO212 as a potential therapeutic candidate for ovarian cancer.

## Additional file

Additional file 1: **Figure S1**. NEO212 promotes apoptosis and mitochondrial dysfunction in OVCAR-3 cells. (A) OVCAR-3 cells were subjected to apoptosis assay using Annexin-V &PI staining. (B) Mitochondria structure in OVCAR-3 cells treated with 100 μM NEO212 or DMSO was observed by TEM. Scale bars: 0.5 μm. Mitochondrial shape and area were measured using Fiji Image J. The results shown are means ± SD; ***p < 0.001. (JPG 647 kb)

## Data Availability

Please contact author for data requests.

## Abbreviations

3MA: 3-methyladenine
Ac-LEHD: Ac-LEHD-FMK
ATM: Ataxia telangiectasia mutated
Baf.A1: Bafilomycin A1
CHEK1/CHEK2: Checkpoint kinases 1 and 2
Cl-CASP9: Cleaved Caspase 9
Cyto C: Cytochrome C
DDR: DNA damage response
DMSO: Dimethyl Sulphoxide
DSBs: DNA double strand breaks
EBSS: Earle’s Balanced Salt Solution
EEA1: Early endosome antigen 1
ERK: Extracellular signal-regulated kinase
H2AFX: H2A histone family member X
JC-1: 5,5′,6,6′-Tetrachloro-1,1′,3,3′-tetraethyl-imidacarbocyanine iodide
LAMP1/2: Lysosome-associated membrane protein1/2
MTG: Mito-Tracker Green
NEO212: Temozolomide-perillyl alcohol conjugate, TMZ-POH
NPC: Human nasopharyngeal carcinoma
NSCLC: Non-small cell lung cancer
PI: Propidum Iodide
POH: Perillyl alcohol
Pro-CASP3: Pro-Caspase3
ROS: Reactive oxygen species
TEM: Transmission electron microscope
TFEB: Transcription factor EB
TMZ: Temozolomide
TNBC: Triple-negative breast cancer
Z-VAD: Z-VAD-FMK
