# Supplementary figures and images for: NEO212 induces mitochondrial apoptosis and impairs autophagy flux in ovarian cancer
Source: J Exp Clin Cancer Res. 2019 Jun 7;38:239. doi: 10.1186/s13046-019-1249-1 (PMC6554966; doi:10.1186/s13046-019-1249-1)

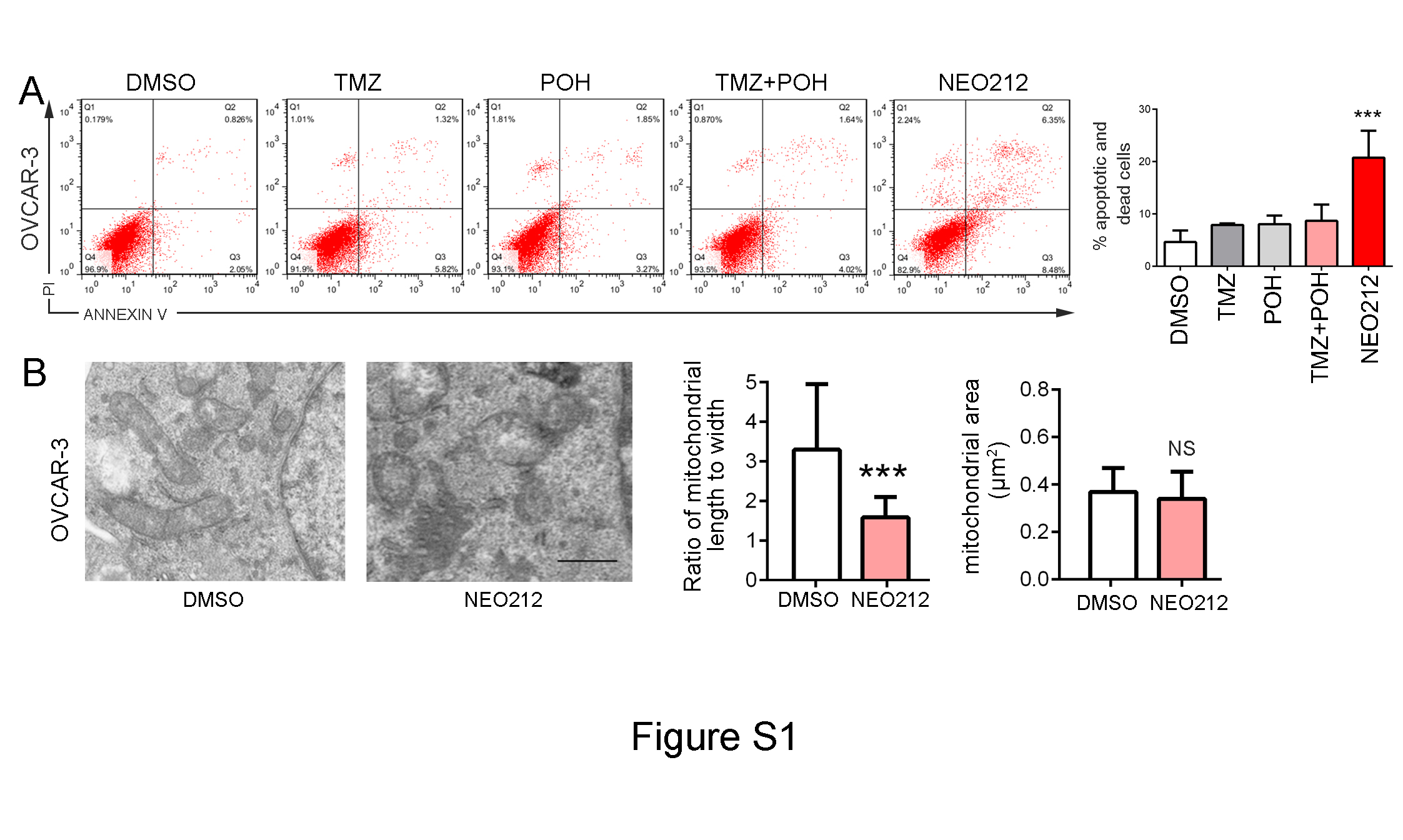

Supplement: Supplementary file 1 — Figure S1. NEO212 promotes apoptosis and mitochondrial dysfunction in OVCAR-3 cells. (A) OVCAR-3 cells were subjected to apoptosis assay using Annexin-V &PI staining. (B) Mitochondria structure in OVCAR-3 cells treated with 100 μM NEO212 or DMSO was observed by TEM. Scale bars: 0.5 μm. Mitochondrial shape and area were measured using Fiji Image J. The results shown are means ± SD; ***p < 0.001. (JPG 647 kb) [file 13046_2019_1249_MOESM1_ESM.jpg]
